# The Relations of the Periosteum to Osteogenesis

**Published:** 1864-10

**Authors:** E. S. Gaillard

**Affiliations:** Surgeon and Medical Inspector.


### Art. IV.—*The Relations of the Periosteum to Osteogenesis.*

By E. S. Gaillard, Surgeon and Medical Inspector.

In the practice and literature of surgery no fact is apparently more frequently accepted as truth than that the periosteum is necessary to osteogenesis; that it is indespensable for the vitality and reparation of bone.

By many of our respectable authorities, both in the library and in the lecture-room, this doctrine is, however, taught, and we are told that where bone is deprived of its periosteum it must die; that in such an event, it is the part of good surgery to anticipate nature in its removal.

It is not unusual at our operating tables to witness the removal of fragments of bone which, though stripped of their periosteal investment, have yet their histologic connection with the medullary structure uninjured; to see the chain-saw and bone-pliers applied to the extremities of bone which had been denuded of their periosteum.

If it can be demonstrated that the fragments thus removed would have soon co-operated in re-establishing osseous integrity, and that the ends of bones so mutilated would soon have been recovered with periosteum and restored by nature to their normal condition, it is evident that much of the surgical interference, now so frequently instituted, would be justly condemned, as injurious and unnecessary.

As in the animal economy "the blood is the life thereof," of course neither the original formation of bone, nor its reparation can occur without a physiologically complete circulation; if any tissue be deprived of its blood, either by obstruction of the circulation or as the result of violence, it must die.

The death of bone, when denuded of its periosteum, is attributed to this ostensible cause, that bone, for its growth, vitality and reparation, is dependent upon the blood obtained through the vessels of the periosteum. Is this a physiological truth?

It is of course known to every tyro in anatomy that the arteries distributed to bones are usually divided into three classes: the first class is that which is supplied from the periosteum; these vessels are small in character and indefinite in number, penetrating the cancellated structure of the bone, and inosculating, by their radicles, with the capillaries of the arteries of the second and third class. The arteries of the second class penetrate directly by foramina, the extremities of the long bones: in other bones they enter at no common point, but irregularly. The third class consists of the nutrient artery penetrating, by its proper foramen, near the centre of the bone.

"The arteries of the two first classes are generally extremely small and ramify upon the compact and cellular structure, penetrating it in every direction and anastomose with the radicles of the nutrient artery."—*Horner.*

It is thus evident that the radicles of the nutrient artery freely anastomose, as well with the capillaries of the arteries penetrating the ends of the bones; as with those of the arterie derived from the periosteum and that in the event of the periosteum being destroyed, the cancellated structure of the bone is fully supplied with blood, from the anastomosis existing between the radicles of the nutrient artery and the capillaries of the arteries penetrating the bones at their ends; or as may be more clearly and now briefly stated, the cancellated structure of the bone is, for its vitality and reparation, independent of the supply of blood derived from the arteries of the periosteum.

These facts are, of course, known to every student of anatomy; the only subject of uncertainty being in regard to the completeness of this capillary inosculation and its supplemental adequacy for sustaining the vitality of the cancellated structure, when the periosteal vessels have been ruptured. Several writers have contended that this anastomosis is entirely sufficient for the objects to be accomplished.

Mr Cooper was of the opinion that "the external and internal periosteum bear strict resemblance to the cellular neurilemma of a nerve, to the membranous covering of the sarcolemma of a muscle and the parenchyma of the various viscera, each being for the same purpose, that of forming a nidus or basement membrane for the products eliminated from the blood." He also states that the importance of the medullary circulation, in the formation of bone, "may be proved by destroying the medullary membrane in the bone of a living animal, when the inflammation which is consequently set up, extends to the external periosteum;" showing thus the intimate connection existing not only between the medullary membrane and the periosteum, but between the medullary membrane and the cancellated structure, which is of course the medium of communication.

It is an important fact that Fleurens, in his experiments with madder, for determining the mode of histologic nutrition, observed that the coloring of bone was not centripetal in its tendency (showing thus the preponderance of the periosteal circulation) but that it was uniformly diffused, proving that bone derived its vitality and nutrition, as well from the medullary circulation, as from the vessels of the periosteum.

Craigil, in his elements of general and pathological anatomy, states that "though the periosteal vessels are the main agents of ossification originally, there is reason to believe that the medullary vessels contribute to its growth and nutrition, after it is formed. This may be inferred from the phenomena of fractures and of those experiments in which the medullary membrane is injured."

Collateral evidence, tending to prove that the periosteal investment is not essential to the production of bone, may be obtained from the phenomena of intra-uterine osteogony.

Many large and important muscles are, by extensive surfaces, attached directly to bone, no periosteum intervening and no evidence of its having intervened or been instrumental in the formation of such portions of these bones. These facts may be observed in the attachments of the quadriceps femoris, pectoralis major, deltoid, the latissimus dorsi, glutaei, coraco-brachialis, triceps, gastrocnemii, etc.; and also in the attachments of many ligaments; ligamentum patellæ, ilio-sacral, interosseous ligaments and the ligamenta subflava.—(Peaslee's Histology.)

It is thus evident, that, in intra-uterine life, bone is formed where the periosteum does not exist, and is independent of the periosteal vessels for its vitality and growth.

Though the medullary membrane has not been satisfactorily proved to exist in fœtal life (Craigil) it is from the vessels forming, subsequently, the basis of this membrane that bone is developed and supported, where the periosteum is absent.

Is bone in extra-uterine life equally independent of the periosteum, in the event of injury or violence sustained by this membrane?

Peaslee, in his recent work on histology, asserts "that the question whether the plasma, from which the new osseous tissue is developed, is exuded by the periosteum or by other parts, is not as important as some authors seem to have held. Obviously, it makes no difference whether it be poured out by the vessels of the periosteum alone, (as it certainly is, in part,) or not. Whencesoever derived, however, it can be organized into bone, whilst in contact with bone. Hence the importance of leaving all the spiculæ of bone (whether denuded of the periosteum or not) as centres of ossification, provided they are not so detached as to act as foreign bodies."

As satisfactory as is this evidence of the independence of bones (in their reparation) in regard to their periosteal investments, the testimony of Dr. Horner is still more complete.

This writer states that "some physiologists have attempted to give to the periosteum the exclusive credit of the formation of callus. This view is erroneous, because experiments show that even where the periosteum is stripped designedly from the fractured ends of bones, they nevertheless unite, and the periosteum is restored when the callus is formed." This testimony is particularly satisfactory, for it not only proves that bones denuded of their periosteum will unite independently of their periosteum, but that the periosteum is only re-developed *after* callus has been formed, and is consequently *not the agent of reparation.*

"The probability is that all the blood-vessels (from whatever source they come) which penetrate the organized coagulated lymph, exuded between the ends of fractured extremities, convey and deposit calcareous matter."—*Horner.*

In a lecture on conservative surgery, delivered at the Royal College of Surgeons, June, 1864, by Mr. William Ferguson, F. R. C. S.; F. R. S.; Professor of Surgery at this college, the following interesting testimony is given; the high position which this gentleman justly fills entitling his opinions to entire respect and confidence: "I believe it to be a common opinion that when a piece of bone is bare or a joint grates, there is no probability of recovery in the part, and that amputation is the proper course. This, however, is a great error: for bare bone is covered again in many instances and a joint may be still so far restored that there may be a certain amount of motion in it, if not, there may still be a cessation of disease, with a useful member. Even when bone *is* dead, nature causes a separation and thus leads the way to its removal, either by spontaneous evolution or by the hands of the surgeons, so that a limb may be retained," etc.

As facts are, however, by the practical surgeon more highly esteemed than opinions and deductions, however plausible, a report of an interesting case tending to prove the truth of views herein considered, is now submitted. This case occurred at the General Hospital at Charlottesville, Virginia, in charge of Surgeon J. L. Cabell, through whose courtesy a report of it is herewith presented. The patient was immediately treated by Assistant-Surgeon F. L. Bronaugh in charge of the ward, and subject, throughout the history of the case, to the active supervision of Surgeon J. S. Davis in charge of the division.

Surgeons Cabell and Davis (in connection with Assistant-Surgeon Bronaugh) watched this case with interest and care, and there was on their part an entire unanimity relative to opinions stated in the report. These gentlemen are, of course, intimately familiar with the surgical literature in regard to such a case, and, in common with other high authorities cited, recognize the material fact that bony union is not absolutely dependent upon periosteal integrity.

The history of this case proves that, under the most unpromising circumstances, the ends of bones denuded of their periosteum will unite. It is, so far as has been observed, the only case occurring in this war tending to prove this fact, and one which, in its relations to conservative surgery, is both important and interesting.

"Sergeant W. G. P., company "B.," South Carolina infantry, Kershaw's brigade, etc., was wounded at the Wilderness, May 6th, 1864, and received at this hospital May 12th; age 20; previous health good; by occupation, a farmer. The ball, presumed to be a conical leaden bullet, penetrated right buttock, four inches posterior to trochanter major, and passing obliquely forward and downward, emerged at a point two inches in front and one inch below the trochanter. When admitted the patient complained of great pain, was restless, desponding and feverish, with total want of appetite. The decubitus was left lateral, with right hip elevated and thrown forward. The daily arrival of a large number of cases demanding immediate attention, precluded the possibility of a very careful examination of this case until the 17th of May, at which time the patient was suffering from irritative fever, in an alarming degree. A hard tumor was felt just below the inferior limits of the trochanter, on the outer aspect of the limb; it was supposed to be due to a displaced and separated fragment of the shaft of the bone. The patient was placed under the influence of chloroform, and an incision made over the tumor, when it was ascertained that it was caused by the abduction (with flexion) of the upper fragments. The wound being explored with the finger, it was found that there was oblique fracture, without comminution, of the upper part of the shaft, and that the anterior aspect of the upper fragment was stripped of its periosteum for two inches, viz: to the point of insertion of the capsular ligament. The case was so very unpromising that the question of the application of Smith's anterior splint was discussed, more with a view to affording some temporary relief to the sufferer, than with a hope of ultimate success. The splint was applied on the 18th with the effect of giving immediate and great relief to the patient. The impossibility of overcoming the extreme abduction of the upper fragment rendered it necessary to place the entire limb in a corresponding direction. His improvement was marked in a few days. The inflammatory tumefaction underwent a rapid abatement; the fever subsided and appetite returned. No complication supervened. On the 28th of July a large sequestrum was removed in several pieces. It represented a scale of bone from the anterior aspect of the upper fragment, and doubtless corresponded to the denuded portion above referred to. The splint had been removed on the 19th of July. He remained in bed until September 1st, when he made his first experiment by walking with the aid of crutches. His limb was shortened about two inches."

The importance of the periosteum is of course great, but is often overestimated.

Dr. Toland, of California, first brought to the notice of the profession in 1864, the fact that the phalanges of the fingers would be reformed, if the periosteum be preserved. This truth has been demonstrated in regard to other bones, and Dr. Peaslee, in his histology, states that the maxilla inferior, the costæ, the scapula and the clavicle have all been reformed under these conditions. M. Maisonneuve has recently removed the tibia with the same result.* Heine states that a rudimentary bone has been formed, when the whole periosteum, as well as the bone, had been removed. It must be confessed that this statement is to be regarded as a tentative appeal to the credulity of the profession.

Since the publication of these cases, showing that entire bones may be reformed if their periosteum be preserved, the importance of this membrane has been more than ever exaggerated, and the assumption has been almost unconsciously made, that if necessary to the regenesis of an entire bone, it is equally necessary for the reparation of a part of it.

To sum up all that has been stated in this connection, it is evident that the prognosis which usually obtains, where bones are denuded of their periosteum, is not justified or warranted by recorded facts; that there is no physiological reason for assuming that bone, under such circumstances, will die, but, on the contrary, that it will usually be recovered with periosteum and restored to its normal condition.

If this be the truth, and if it be so recognized, surgical interference in all cases of denuded bone will be abandoned, and one step secured in the progress of conservative surgery.

NOTE.—In connection with the subject of conservative surgery, and in view of the great distrust with which resections of the knee-joint are regarded in our service (on account of the sad fatality which has attended these operations during the last campaign) it may not be inappropriate to briefly present statistics, recently furnished (June, 1864,) by Mr. Ferguson, Professor of Surgery in the Royal College of Surgeons, London.

Mr. Jones, of Jersey, performed this operation 15 times, with one death; Mr. Ferguson 40 times, with 15 deaths; at Exeter Hospital, 12 operations with no deaths; Dr. Humphrey has operated 13 times, with one death; at King's College there have been 10 operations performed, with one death.

Mr Price has collected the records of 250 cases; the mortality in these is not greater than that which obtains in amputation through the lower third of the thigh. Many of these patients walked on crutches in six weeks, and some in three weeks after the operation.

Mr. Parke was the first to perform this operation in surgery; period, 1782.

This patient of Mr. Parke made several voyages to sea, in which he was able to go aloft with considerable agility, and to perform *all* the duties of a common seaman.

These facts are stated that those who (from the records of our service) are skeptical as to the propriety and policy of this operation may be induced to reconsider the subject and save their patients from the terrible mutilation which must otherwise befall them.

When such excellent comparative results are obtained by Confederate surgeons in the other domains of surgery, there is no reason why this field (so far a Golgotha) should be avoided and proscribed.

Acknowledgment is, with pleasure, made to Surgeon A. M. Fauntleroy for many facts presented in this paper.

